# Inhibition of Cell Proliferation by an Anti-EGFR Aptamer

**DOI:** 10.1371/journal.pone.0020299

**Published:** 2011-06-08

**Authors:** Na Li, Hong Hanh Nguyen, Michelle Byrom, Andrew D. Ellington

**Affiliations:** 1 AM Biotechnologies, Houston, Texas, United States of America; 2 Department of Chemistry and Biochemistry, University of California Los Angeles, Los Angeles, California, United States of America; 3 Institute for Cellular and Molecular Biology, University of Texas, Austin, Texas, United States of America; 4 Institute for Cellular and Molecular Biology, Center for Systems and Synthetic Biology, University of Texas, Austin, Texas, United States of America; University of Florida, United States of America

## Abstract

Aptamers continue to receive interest as potential therapeutic agents for the treatment of diseases, including cancer. In order to determine whether aptamers might eventually prove to be as useful as other clinical biopolymers, such as antibodies, we selected aptamers against an important clinical target, human epidermal growth factor receptor (hEGFR). The initial selection yielded only a single clone that could bind to hEGFR, but further mutation and optimization yielded a family of tight-binding aptamers. One of the selected aptamers, E07, bound tightly to the wild-type receptor (K_d_ = 2.4 nM). This aptamer can compete with EGF for binding, binds to a novel epitope on EGFR, and also binds a deletion mutant, EGFRvIII, that is commonly found in breast and lung cancers, and especially in grade IV glioblastoma multiforme, a cancer which has for the most part proved unresponsive to current therapies. The aptamer binds to cells expressing EGFR, blocks receptor autophosphorylation, and prevents proliferation of tumor cells in three-dimensional matrices. In short, the aptamer is a promising candidate for further development as an anti-tumor therapeutic. In addition, Aptamer E07 is readily internalized into EGFR-expressing cells, raising the possibility that it might be used to escort other anti-tumor or contrast agents.

## Introduction

Aptamers have been selected against a surprising range of targets, ranging from ions to small organics to proteins to supramolecular structures such as viruses and tissues [1], [2]. Aptamers targeting proteins in the bloodstream or on cell surfaces have proven to be useful for therapy. For instance, aptamers have been selected against a number of growth factors such as basic fibroblast growth factor (bFGF) [3], vascular endothelial growth factor (VEGF) [4], platelet-derived growth factor (PDGF) [5], and keratinocyte growth factor (KGF) [6]. These aptamers could block the interactions between growth factors and their receptors, and have proven to be excellent drug candidates. An anti-VEGF aptamer has been approved by FDA in 2004 for the treatment of neovascular age-related macular degeneration.

Aptamers are not only useful in their own right, but as escorts for therapeutic or diagnostic reagents. Modified RNA anti-PSMA (prostate-specific membrane antigen) aptamers [7] have been used by many research groups as targeting agents and conjugated to a variety of molecules including gold nanoparticles, siRNA, and drug encapsulated polymer particles for specific delivery [8], [9], [10], [11], [12]. Modified RNA aptamers against the virion surface glycoprotein, gp120 [13] were conjugated to an anti-human immunodeficiency virus siRNA, and both the aptamer and the siRNA portions of the chimera had potent anti-HIV activity [14].

Aptamers targeting cell surface receptors may be amongst the most useful for biomedical applications (reviewed in [15], [16]). HER3 (human epidermal growth factor receptor-3) is membrane-bound protein and is related to the development of some malignant tumors. RNA aptamers against HER3 have shown strong inhibitory effects on hrg (heregulin)-induced growth stimulation of MCF7 cells [17]. Both DNA and RNA anti-mouse transferrin receptor aptamers have been selected and used to mediate the endocytosis of lysosomal enzymes [18]. Anti-RET receptor tyrosine kinase aptamers have been selected against cells expressing human RET, and one of them was found to block RET-dependent intracellular signaling pathways. [19].

We have previously isolated a RNA aptamer targeting EGFR and utilized it for nanoparticle delivery [20]. Here we report a 2′ F-Py modified anti-EGFR aptamer that can inhibit EGF stimulated EGFR phosphorylation and cell proliferation. This aptamer may provide the basis for further development of anti-tumor therapeutics.

## Materials and Methods

### In vitro selection of anti-EGFR 2′-fluoropyrimidine RNA aptamers

The DNA library for selection consisted of a 62-nucleotide random region (N62) flanked by two constant regions: 5′-gataatacgactcactataggcgctccgaccttagtctctg-N_62_-gaaccgtgtagcacagcaga-3′ (T7 RNA polymerase promoter is underlined). The initial RNA pool was generated by transcribing some 10^14^ DNA templates using a Durascribe kit (Epicentre, Madison, WI) followed by DNase treatment and PAGE purification. About 2 nmoles RNA and 90 pmoles recombinant human EGFR-Fc (hEGFR) fusion protein (R&D Systems, Minneapolis, MN) were used for each round of selection in a reaction volume of 100 µL.

To prepare the substrate for selection, human EGFR-Fc protein was immobilized to Protein G magnetic beads (New England Biolabs, Ipswich, MA) as follows: Protein G beads (200 uL) were first washed twice with 200 uL of DPBS (Invitrogen, Carlsbad, CA). Beads were removed from the DPBS buffer and hEGFR (50 µg) resuspended in 200 µL DPBS was added. The immobilization reaction was incubated overnight at 4°C. Protein G beads with or without human EGFR (hEGFR) were washed twice with Selection Buffer (1X DPBS and 5 mM MgCl_2_) prior to being used in selections.

For rounds of selection, RNA was first thermally equilibrated by heating to 75°C for 3 minutes and cooling at 1°C/s to ambient temperature in 100 µL of Selection Buffer, and then incubated with 100 µL of Protein G beads. Following this negative selection, the RNA solution was removed and incubated with the hEGFR-conjugated protein G beads (50 µL) at 25°C for 30 min. The human EGFR-conjugated Protein G beads were washed 3 times with 100 µL of Selection Buffer and then heated to 95°C for 5 min in Elution Buffer (200 mM NaCl, 25 mM EDTA, and 8 M urea) to release any bound RNA. The eluted RNA was rinsed over M30 filters (Millipore, Bedford, MA) twice with 150 µL of water to remove salt and urea, eluted in water, reverse transcribed, and PCR amplified. The DNA pool from Round 10 of the selection was cloned and sequenced according to standard procedures.

Aptamer E01 was resynthesized as a 30% doped sequence pool as previously described [20], and used for selection. The selection with the doped pool was carried out as above, except that RNA was first incubated with hEGFR-conjugated Protein G beads. Eluted RNA was purified using M30 filters and then incubated with hIgG (human IgG, R&D Systems)-conjugated Protein G beads at 25°C for 30 min. Human IgG-conjugated Protein G beads were prepared as described above for hEGFR-conjugated Protein G beads. RNA that remained in solution following this negative selection was again purified using M30 filters, reverse transcribed, and PCR amplified. The DNA pools from Round 7 (30 clones) and Round 9 (40 clones) were cloned and sequenced.

### Binding specificities and dissociation constants

To make monomeric hEGFR, about 0.3 µg of hEGFR was incubated in Selection Buffer with or without 5 mM DTT for 10 min at 25°C. To confirm that the monomer had been produced the solution was mixed with 4X loading dye and loaded onto a 4–12% NuPAGE gel (Invitrogen) with 1X MOPS running buffer alongside marker proteins. The gel was developed at 200 V for 1 hour, and stained as previously described (Supplementary Material, [20]).

Aptamers E03, E04, and E07 were assayed for their ability to bind either dimeric or monomeric hEGFR. Some 10 nM [α-^32^P]-ATP-labeled (3000Ci/mmol, 10mCi/ml, Perkin Elmer, Waltham, MA) Aptamers E03, E04, and E07 were incubated with 100 nM hEGFR (with or without DTT treatment) for 30 min at 25°C in Selection Buffer. To assess binding specificity, about 10 nM of [α-^32^P]-ATP-labeled Aptamers E03, E04, and E07 were also incubated with 100 nM hIgG and mEGFR (mouse EGFR) with or without DTT treatment for 30 min at 25°C in Selection Buffer. All protein were from R&D Systems. The binding reaction was loaded onto on a vacuum manifold (Schleicher & Schuell, Keene, NH) with two layers of filters. The top layer was nitrocellulose and captured only the RNA:protein complexes, while the bottom filter was nylon and captured all remaining RNA. Sample wells were washed three times with 300 uL of Selection Buffer, and nitrocellulose and nylon filters were dried and visualized using a Phosphorimager (GE Healthcare, Piscataway, NJ).

To measure dissociation constants for aptamer:protein complexes, about 0.1 nM [γ-^32^P]-ATP-labeled Aptamers E03, E04, and E07 were incubated with different concentrations of hEGFR (0.1 nM, 0.3 nM, 1 nM, 3.2 nM, 10 nM, 32 nM, and 100 nM) and mEGFR (1 nM, 3.2 nM, 10 nM, 32 nM, and 100 nM, 316 nM, and 1000 nM) for 30 min at 25°C. The binding assays were carried out as described above, and dissociation constants were calculated as previously describe [20].

### Assaying cell surface binding of anti-EGFR aptamers

Aptamers were synthesized with a 24 nt extension at 3′ end (5′- GAAUUAAAUGCCCGCCAUGACCAG-3′) and hybridized to a biotinyated DNA oligoucleotide. Phycoerythrin-labeled streptavdin (SA-PE, Prozyme, San Leandro, CA) was added to the RNA:DNA duplex without further purification [15].

A431 cells were purchased from ATCC (American type Culture Collection, Manassas, VA) and MDA-MB-435 cells were obtained from the laboratory of Dr. Konstantin Sokolov at University of Texas at Austin. Both cell lines were cultured in DMEM (ATCC) with 10% FBS (Invitrogen). Cells were grown to 70% confluence, trypsinized, washed, and counted.

About 0.2 million cells were incubated with 100 nM labeled pools or aptamers in 100 uL of Selection Buffer for 30 min at 25°C. Cells were then washed with 100 uL of Selection Buffer 3 times and resuspended in 300 uL of Selection Buffer. Samples were analyzed on the FL2-H channel of a FACSCalibur (BD Biosciences, San Jose, CA).

Competitive binding to the cell surface was assessed by mixing Alexa Fluor 488-labeled EGF (0.1 ug/mL, ca. 1.5 nM, Invitrogen) with either unselected N62 pool RNA pool, Aptamer E03, Aptamer E04, or Aptamer E07 (1 uM ). The competitive binding reactions were incubated with 0.2 million trypsinized and washed A431 cells in 100 uL of Selection buffer at 25°C for 30 min. Samples were washed 3 times with 100 ul of Selection Buffer, resuspended in 300 ul of Selection Buffer, and analyzed on the FL1-H (for Alex Fluor 488) channel of a FACSCalibur.

### Tyrosine phosphorylation assay

A431 cells were seeded in a 24-well plate, grown for 24 hours in DMEM medium with 10% FBS, and then serum-starved for 18 hours. Cells were then incubated for 20 min at 37°C with either 2 nM EGF,, 2 nM EGF and 100 nM Ab C225,, 2 nM EGF and 1 µM unselected N62 pool RNA,, 2 nM EGF and 1 µM Aptamer E07,, 1 µM unselected N62 pool RNA,, or 1 µM Aptamer E07. After removing the media, cells were lysed in 100 µl 1X RIPA buffer (Thermo Fisher Scientific, Waltham, MA) for 5 min and further disrupted using an ultrasonic dismembrator (Thermo Fisher Scientific). Western blot analysis was done as previously described [15]. A biotinylated anti-phosphotyrosine antibody (4G10) (Millipore) was used as the primary antibody and an anti-mouse IgG-AP conjugate (Promega, Madison, WI) was used as the secondary antibody.

### Internalization assay

The internalization of Aptamer E07 was assayed by flow cytometry as previously described (Supplementary Material, [20]). A negative control (Mutant Aptamer) was generated by scrambling the sequence derived from the random region of Aptamer E01 (http://workbench.sdsc.edu/). Phycoerythrin-labeled Aptamer E07 and the Mutant Aptamer were incubated with 0.2 million trypsinized and washed A431 cells for 30 min either on ice or at 37°C in 100 uL of Selection Buffer. The cells were then treated with 0.01 u/µL Riboshredder (Epicentre Biotechnologies, Madison, WI) for 10 min at 25°C followed by washing 3 times with 100 µL of Selection Buffer. Samples were resuspended in 300 ul of Selection Buffer and analyzed by flow cytometry as described above. The amount of internalized Aptamer E07 was calculated using the following equation:

*RNA Internalized*

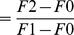
(1)


where F0, F1, and F2 represent the fluorescence of Mutant Aptamer-labeled cells, the fluorescence of Aptamer E07-labeled cells, and the fluorescence of Aptamer E07-labeled cells after Riboshredder treatment, respectively.

### Cell proliferation assay in 3D culture

A431 cells were trypsinized and resuspended in Matrigel (BD Biosciences). On day 0, about 3,000 cells in 200 ul of Matrigel were seeded in a 48-well plate and covered with 200 ul of complete media (DMEM with 10% FBS). The media was replaced on Days 1, 3, 5, 7, 9, and 11 with 200 ul of DMEM with 1% FBS containing either 1 uM Mutant Aptamer, Aptamer E07, dephosphorylated Mutant Aptamer or dephosphorylated Aptamer E07. On Day 13, media -was removed and the Matrigel was incubated with 400 ul of Cell Recovery Solution (BD Biosciences) on ice for 4 hours. Samples containing A431 colonies were transferred to a 24-well plate and imaged with an IX51 Inverted Microscope (Olympus, Center Valley, PA) under 4x objective. Released cell colonies were lysed and the nucleic acids content was measured using a CyQUANT® Cell Proliferation Assay Kit (Invitrogen). Results were analyzed in Microsoft Excel with Anova analysis.

Dephosphorylated aptamers were prepared by incubating RNA with Antarctic Phosphatase (New England Biolabs, Ipswich, MA). Some 8 nmoles of RNA was incubated with 75 units phosphatase in a 100 uL reaction at 37°C for 30 min. The phosphatase was deactivated at 65°C for 5 min. RNA was purified prior to use by ethanol precipitation.

### Assaying binding of aptamer E07 to the EGFRvIII deletion variant

To test for binding of Aptamer E07 with hEGFRvIII mutant purified protein, 10 nM [α-^32^P]-ATP-labeled Aptamer E07 was incubated with 50 ug hEGFR (R&D Systems) and 50 ug hEGFRvIII deletion mutant protein (gift of Dr. George Georgiou, University of Texas at Austin), for 30 min at 25°C. The binding assay was carried out as described above.

To test for binding of the aptamer to the deletion mutant in the context of the cell surface, U87MG delta vIII cells were obtained from Dr. Frank Furnani, University of California San Diego, and were cultured in High Glucose DMEM with 10% FBS. Cells were grown to 70% confluence, trypsinized, washed, and counted. FACs assays for cell surface binding and internalization with Aptamer E07 were performed as described above.

## Results

### Isolation of 2′-fluoropyrimidine modified anti-EGFR aptamers

We had previously isolated RNA aptamers against EGFR [20], but now wanted to generate aptamers that would be much more stable *in vivo*. We initially targeted a purified Fc-EGFR fusion protein conjugated to Protein G beads, and initiated selections with a 2′-fluoropyrimidine modified RNA pool that spanned 62 random positions. The *in vitro* half life of 2′-fluoropyrimidine modified aptamers in plasma is typically several hours to days, [21], [22]which should assist with further therapeutic development of any aptamers found. Negative selections against Protein G beads were carried out prior to each round of positive selection. After 10 rounds of selection and amplification the percentage of bound RNA that bound to Fc-EGFR (1 uM) increased from 2% to 39%. Some 33 clones from the final round of selection were sequenced (**Table S1**). Many of the selected sequences contained the consensus motif 5′-GGUGCU-3′ which is known to bind to the Fc portion of the fusion protein (Miyakawa, S., Y. Nomura, et al. 2008). However, one clone, Aptamer E01 (which was isolated 4 / 33 times), did not contain this motif and bound specifically to human EGFR with a dissociation constant of about 40 nM. The random region of Aptamer E01 was only 51 nt in length (rather than 62 nt). This was likely due to the accumulation of a deletion variant during PCR amplification. This aptamer was re-synthesized as a doped sequence pool and the negative selection against human IgG1 was carried out each round after (rather than before) positive selection against human EGFR. After 9 rounds of selection, the percentage of bound RNA that bound to Fc-EGFR increased from 2% to 22%. The selected pool also showed some cross-binding to the non-cognate protein Fc-ErbB2 (5%), but this is perhaps not surprising as the original unselected N62 pool showed very high background binding to hErbB2 (32%). This background binding may be due to the positively charged polyhistidine tag on hErbB2, which is not present on hEGFR.

Twenty one clones from Round 7 and 51 clones from Round 9 were sequenced (the sequences derived from the random regions are shown in **Table S2**). While the wild-type aptamer was not recovered, Aptamer E30 and E39 appeared twice, and all other aptamer sequences appeared once.

Because of the sequence diversity of isolated aptamers, they were further screened for their ability to bind to cells expressing EGFR. Aptamers were transcribed with a 24-nt extension, hybridized with a biotinylated antisense oliognucleotide, and incubated with SA-PE. Labeled aptamers were incubated with A431 cells, and binding was analyzed by FACS. Aptamers E02-, E03-, E04-, E05-, E06-, and E07-labeled cells showed greater fluorescence signals than other aptamers (**Table 1**). Interestingly, these aptamers all contained U40G and C67A mutations which could reinforce a particular aptamer conformation (**Figure S1**). When comparing the parental aptamer (E01) and the derived aptamer E07, G40 reinforces a predicted stem, while A67 disfavors a short stem and reinforces an internal loop.

**Table 1:** Sequences of isolated anti-EGFR aptamers.

Clone	Sequence
E01	UGCCGCUAUAUCGCACGUAUUUAAUCGCCGUAGAAAAGCAUGUCCAAGCCG
E02	UG*G*CGCUA*A*AU*A*GCACG*G*A*AA*UAAUCGCCGUAGAAAAGCAUGUC*A*AAGCCG
E03	UGC*UAG*UAUAUCGCACG*G*AUUUAAUCGCCGUAGAAAAGCAUGUC*A*AAGCCG
E04	UGCCGC*C*AUAUC*A*CACG*G*AUUUAAUCGCCGUAGAAAAGCAUGUC*A*AAGCCG
E05	U*U*CCGCU*G*UAU*AA*CACG*G*A*C*UUAAUCGCCGUAG*U*AAAGCAUGUC*A*AAGCCG
E06	UG*U*CGCU*C*UAU*U*GCACG*G*AUUUAAUCGCCGUAGAAAAGCAUGUC*A*AAGCCG
E07	UGCCGCUAUA*AU*GCACG*G*AUUUAAUCGCCGUAGAAAAGCAUGUC*A*AAGCCG

Only the random sequence portions of the aptamers are shown. Substitutions relative to Aptamer E01 are shown in bold italics.

### Aptamer binding specificity and affinity

**Figure 1:**
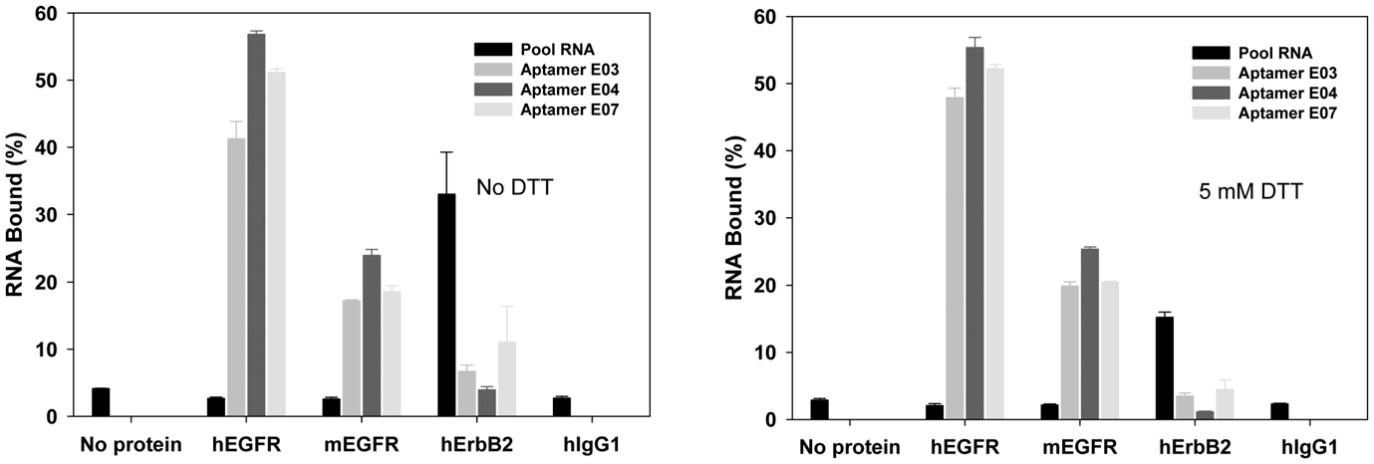
Binding specificity of anti-EGFR aptamers. The N62 pool and aptamers E03, E04, and E07 were assayed in triplicate by filtration for binding to hEGFR, mEGFR, hErbB2, and hIgG1. Average values and standard deviations are shown. Binding assays were carried out either in the absence (left) or presence (right) of DTT. A no protein control was also carried through the procedure. Percent binding was relative to the total RNA added.

**Figure 2:**
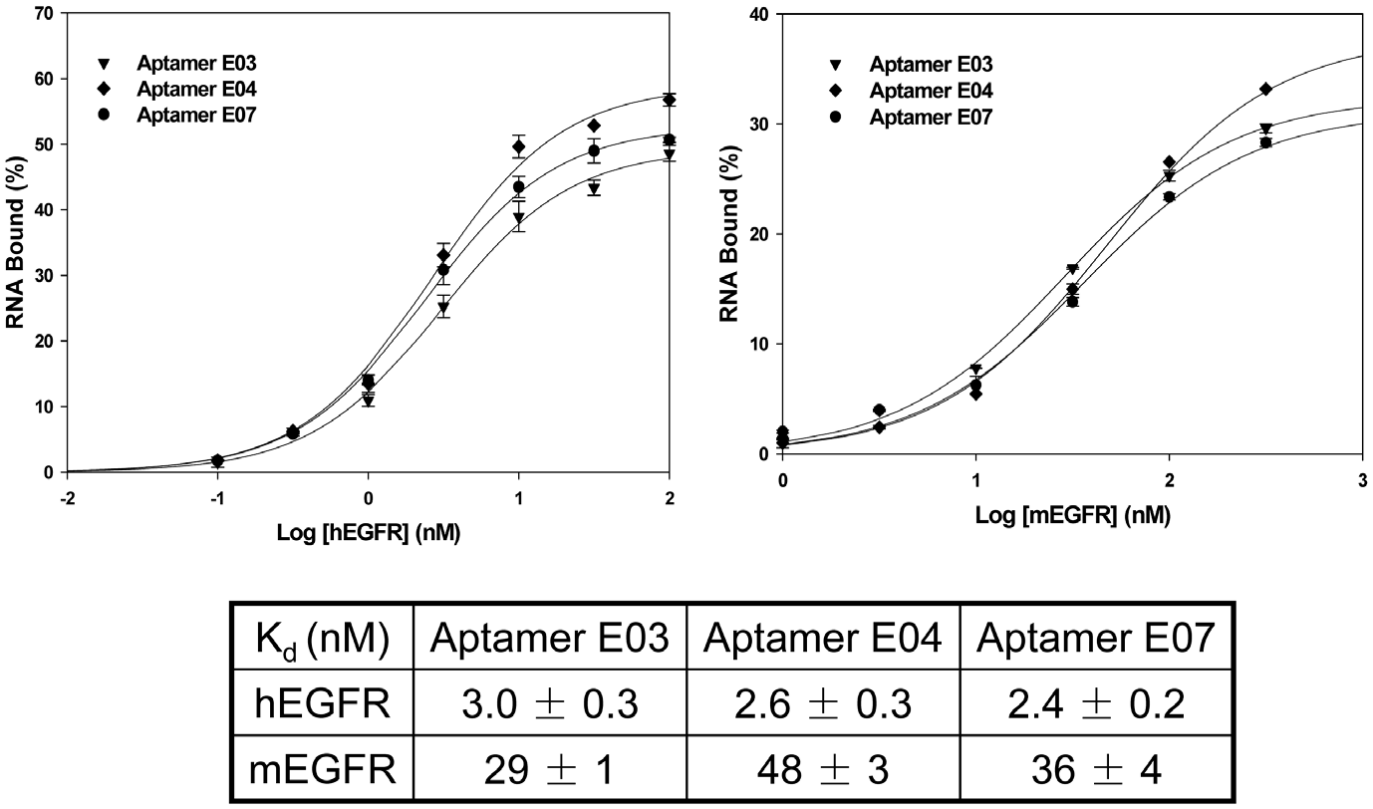
Binding constants for Aptamers E03, E04, and E07. Binding isotherms were constructed using 0.1 nM aptamer and varying amounts of hEGFR or mEGFR. Binding assays were carried out in triplicate and the average value and standard deviation are shown. The fact that binding does not reach 100% is a function of the filtration assay, and is commonly observed. Dissociation constants were calculated following curve-fitting, as described in **Materials and Methods**.

Based on the sequencing and binding assay results, aptamer E03, 04, and E07 were chosen for further characterization. In order to ascertain the specificity of the aptamers, binding to hEGFR, mEGFR, hErbB2, and hIgG1 was probed. As shown in **Figure 1**, these three aptamers bound to both hEGFR and mEGFR, but not to hErbB2 nor hIgG1. The doped sequence selection therefore eliminated the cross-binding to hErbB2 that was observed with the original pool. Cross-binding between the human and mouse forms of the protein is perhaps not surprising because these two proteins show 88% identity in their amino acid sequences. A filter-binding assay was used to generate binding isotherms and the dissociation constants of Aptamers E03, E04, and E07 were found to be 2–3 nM with hEGFR and 30–50 nM with mEGFR (**Figure 2**). The aptamers bound almost as tightly to EGFR as the natural ligand EGF and the therapeutic mAb C225 (both at ca. 1 nM) [23].

Since the selection was carried out against a dimeric form of the protein (dimerization was through disulfide bond formation in the Fc fusion, and this may be be very different from the dimer found on the cell surface), we attempted to determine whether the aptamers could specifically recognize the EGFR dimer. DTT was added to reduce the disulfide bonds and generate an EGFR monomer fusion protein; the formation of monomers was confirmed by native gel electrophoresis as shown previously [20]. As shown in **Figure 1**, the addition of DTT to the binding buffer does not affect the binding affinity of Aptamer E03, E04, and E07.

### Anti-EGFR aptamers bind to cells expressing EGFR

Due to the potential conformational differences between purified proteins and those that reside on cell surfaces, aptamers isolated against purified proteins do not always bind to cells [24]. Therefore, aptamers were assayed for their ability to bind to both A431 epidermoid carcinoma cells (1–3 million EGFR molecules per cell) and MDA-MB-435 breast cancer cells (EGFR deficient). These cell lines had previously been used to detect the cellular binding of a previously selected anti-EGFR RNA aptamer (J18) [20].

**Figure 3:**
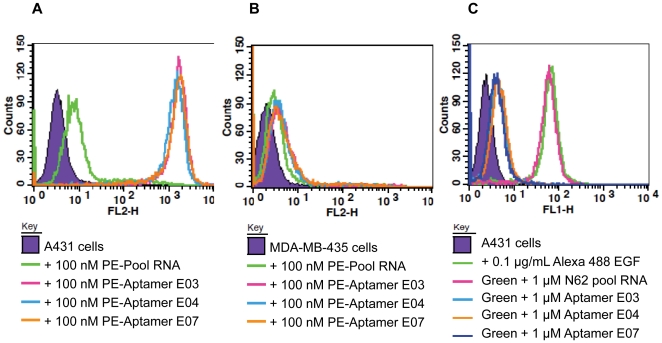
Cellular binding and inhibition of EGF-binding by anti-EGFR aptamers. Phycoerythrin-labeled N62 pool (green line), Aptamer E03 (pink line), Aptamer E04 (cyan line), and Aptamer E07 (orange line) were incubated with EGFR-overexpressing cells (A431); (A) and EGFR-negative cells (MDA-MB-435); (B) and analyzed on the FL2-H channel of a FACSCalibur. A no RNA control was also carried out. Alexa 488-labeled EGF (0.1 ug/ml, ca. 1.5 nM) was incubated with A431 cells (green line), and binding assessed by FACS. The interaction could be blocked by the further addition of 1 uM Aptamer E03 (cyan line), Aptamer E04 (orange line) and Aptamer E07 (dark blue line) but not unselected N62 pool RNA (pink line)(C). Counts represent number of cells counted.

Aptamers E03, E04, and E07 were all found to bind A431 cells but the unselected N62 pool RNA did not (**Figure 3A**). None of the aptamers bound above background to the negative control cell line, MDA-MB-435 (**Figure 3B**). Thus, the aptamers appear to be capable of recognizing monomer EGFR in the context of the cell surface. As was the case with the protein *in vitro*, the addition of DTT did not impact binding to cells.

### Aptamer E07 blocks EGF binding to EGFR and inhibits EGF-stimulated EGFR phosphorylation

**Figure 4:**
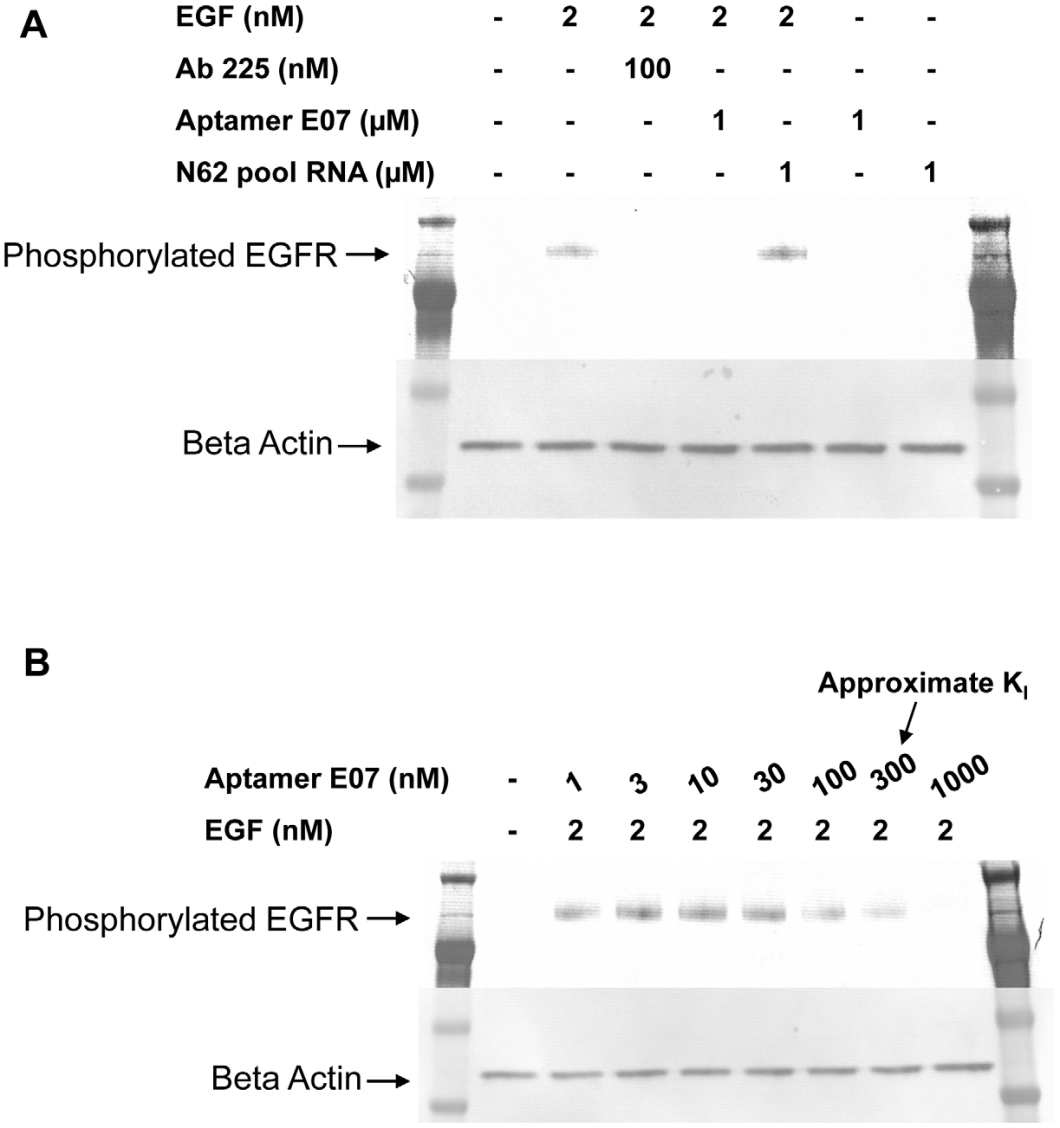
Aptamer inhibition of EGFR phosphorylation. The phosphorylation of EGFR was stimulated by 2 nM EGF and was detected by Western blot analysis using an anti-tyrosine phosphorylation antibody, (A, lane 2). The additions to individual reactions are shown above the gel lanes. Arrows show the position of proteins. Staining was also carried out with an anti-beta-actin antibody to ensure that similar amounts of samples were loaded. Phosphorylation is inhibited by the addition of 100 nM Ab C225 (A, lane 3), 1 uM Aptamer E07 (A, lane 4), but not unselected N62 pool RNA (A, lane 5). Aptamer 07 and pool RNA alone do not induce EGFR phosphorylation (A, lanes 6 and 7). The approximate inhibition constant for Aptamer E07 is about 300 nM (B).

EGF binds to EGFR, and stimulates its dimerization, phosphorylation, and downstream signaling. Efficiently blocking the interaction between EGF and EGFR could inhibit cell proliferation and tumor growth. Using labeled EGF and FACS as an assay, aptamers E03, E04, and E07 were found to block binding of EGF to A431 cells, while the unselected N62 pool RNA did not (**Figure 3C**). Western blot analyses also show that Aptamer E07 blocked EGF-induced EGFR phosphorylation (**Figure 4A**). Different concentrations of Aptamer E07 were used to inhibit EGFR phosphorylation and the inhibition constant was found to be approximately 300 nM (**Figure 4B**). The significant difference between the observed dissociation constant of Aptamer E07 with EGFR and its apparent inhibition constant for phosphorylation inhibition is likely due to the fact that the binding constant was measured against purified protein while the inhibition constant was determined via a cell-based assay.

### Internalization of Aptamer E07

Receptor-mediated endocytosis is a process by which cells uptake molecules, including cytokines such as EGFR, from their surroundings. We have previously developed a flow cytometry-based assay to monitor the internalization of aptamers that bind to receptors on the cell surface [15]. In short, aptamer:PE complexes are added to cells either at 37°C (where internalization is active) or 4°C (where internalization is dormant). After allowing the aptamers to internalize, the cell surfaces are challenged with nucleases. Only those aptamers that have been internalized remain and can be detected by FACS. As a control for internalization, a Mutant Aptamer was designed by scrambling the random region of Aptamer E07.

**Figure 5:**
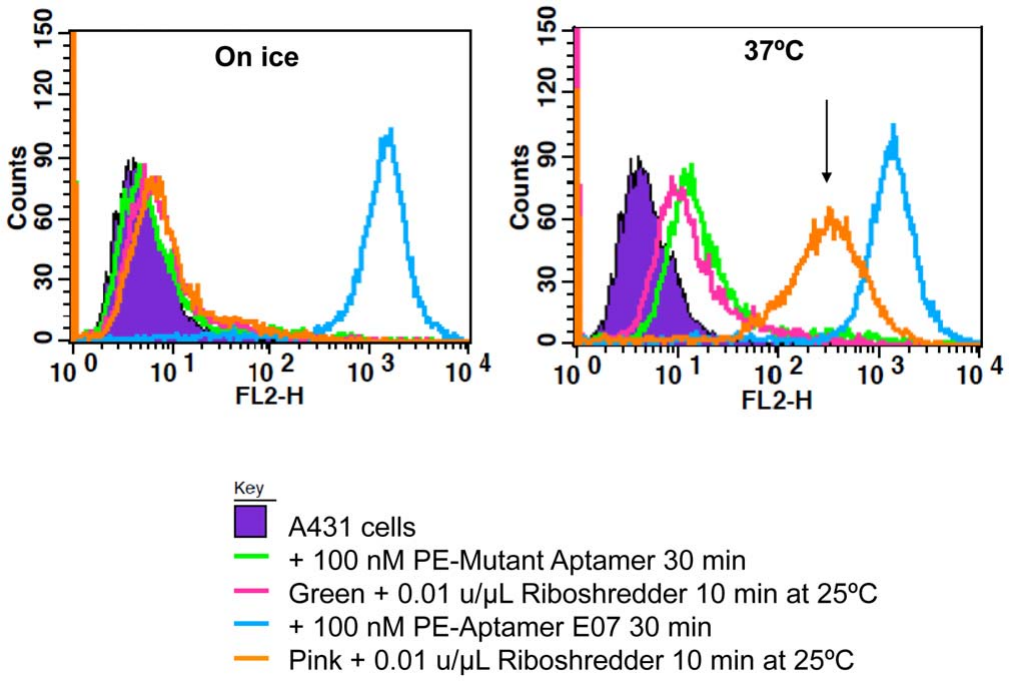
Internalization of anti-EGFR Aptamer E07. Phycoerythrin-labeled unselected N62 pool RNA (100 nM, green line) and PE-labeled Aptamer E07 (100 nM, cyan line) were incubated with A431 cells either on ice (left) or at 37°C (right) for 30 min. After the binding reaction, cells were exposed to Riboshredder for 10 min at 25°C (pink line and orange lines, respectively). Residual fluorescence was analyzed by FACS. Putative internalized Aptamer E07 conjugates are indicated by arrows. Counts represent number of cells counted.

Aptamer E07 and Mutant Aptamer were assayed for internalization. Mutant Aptamer does not bind to A431 cells at either temperature. Riboshredder completely removed Aptamer E07 from A431 cells that were incubated on ice (**left,** **Figure 5**), but about 23% Aptamer E07 was resistant to Riboshredder (**right,** **Figure 5**) at 37°C, presumably because it had been internalized.

### Aptamer E07 inhibits cell proliferation in 3D cultures

**Figure 6:**
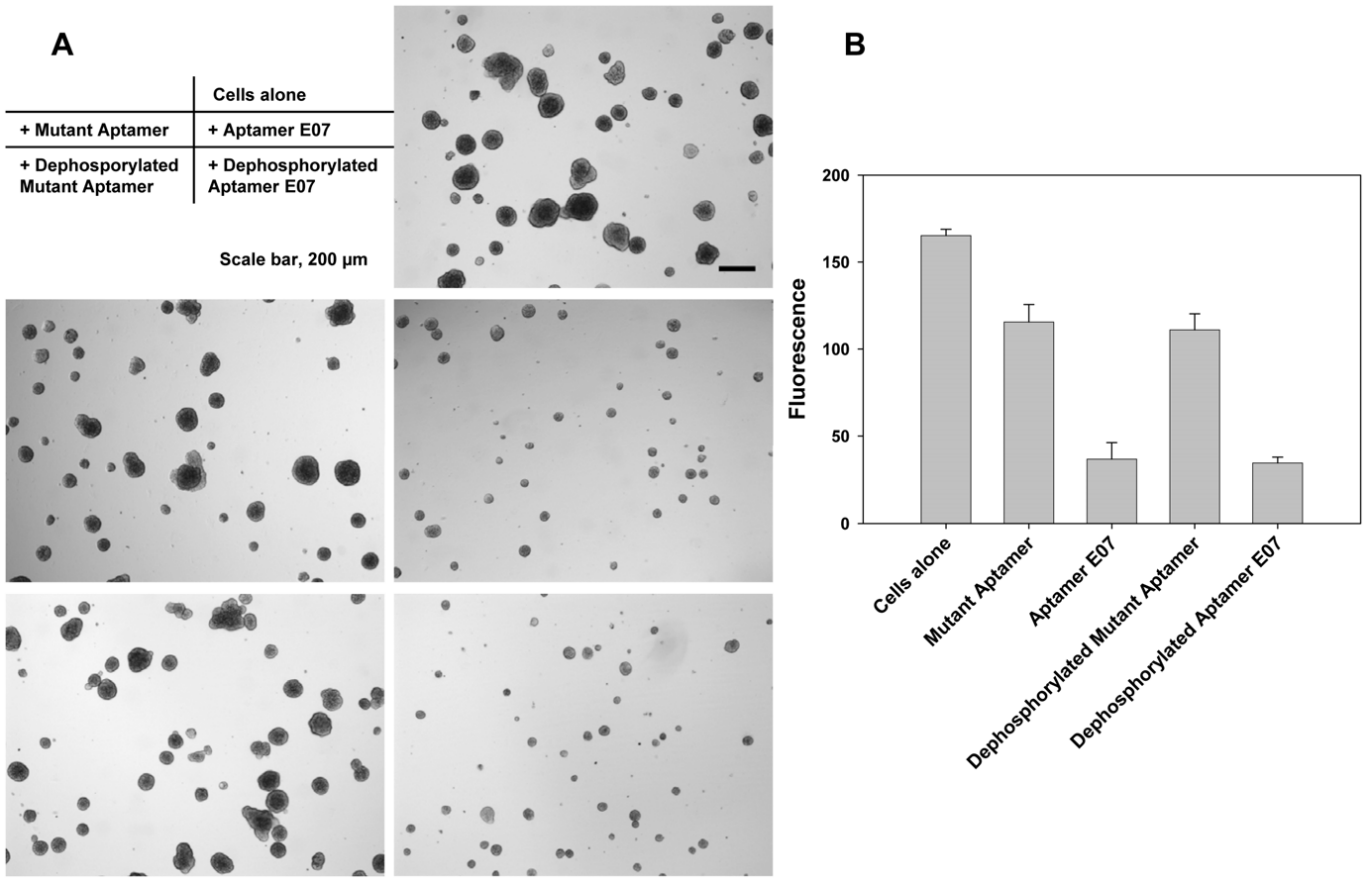
Inhibition of A431 cell proliferation by Aptamer E07. A431 cells were seeded into Matrigel in 48 well plates and treated with untreated or dephosphorylated Aptamer E07 or Mutant Aptamer (1 uM) every other day for a total of 6 treatments. (A) Cell colonies were released from Matrigel by Cell Recovery Solution, transferred to a 24-well plate, and imaged with an IX51 Inverted Microscope (Olympus, Center Valley, PA) under 4x objective. The inset shows which of the five micrographs go with which specific treatment parameters. (B) The nucleic acids content was measured using a CyQUANT® Cell Proliferation Assay Kit. CyQUANT® GR dye was incubated with cell lysate and exhibited strong green fluorescence when bound to cellular nucleic acids. The fluorescence is linearly correlated with the number of cells and readily detected by a plate reader. (p = 5.4E−13).

We evaluated the ability of Aptamer E07 to inhibit A431 cell proliferation. Because culturing cells on flat plasticware results in artificial, two-dimensional sheets of cells, we have grown A431 cells in three-dimensional environments that better mimic their *in vivo* counterparts. To avoid the potential interferon induction by the triphosphate group at the 5′ end of the RNA [25], both Aptamer E07 and Mutant Aptamer were first treated with phosphatase. Dephosphorylation did not impact the binding of E07 to cells (**Figure S2**). A431 cells were treated with Aptamer E07 and Mutant Aptamer every other day for 6 total treatments. The size of the A431 colonies treated with both Aptamer E07 and dephosphorylated Aptamer E07 decreased greatly (**Figure 6A**). Because cell DNA content for individual cells is constant, the amount of DNA measured by the fluorescence intensity correlates with cell number, therefore cell proliferation. The number of A431 cells in the observed colonies decreased about 80% when treated with Aptamer E07 and dephosphorylated Aptamer E07. In contrast, the number of cells decreased about 30% when treated with Mutant Aptamer or dephosphorylated Mutant aptamer. (p = 5.4E−13) (**Figure 6B**). Despite aptamer dephosphorylation, the decreased fluorescence signal of Mutant Aptamer treated cells could be due to non-specific inhibitory effects, such as the activation of innate immune responses, as has been observed with structured siRNAs [26], [27].

## Discussion

The four members of the epidermal growth factor receptor family have proven to be excellent targets for cancer therapy [28]. Aptamers against EGFR members may therefore prove to be excellent therapeutic candidates. Anti- Her3 aptamers have been isolated and have been shown to inhibit heregulin signaling [17]. However, these anti-Her3 aptamers did not contain any modifications and were nuclease sensitive, and thus their *in vivo* application was greatly limited. We have previously isolated an anti-EGFR RNA aptamer [20], that was similarly labile. Nuclease degradation of aptamers can be slowed from a half life of only a few minutes to hours by incorporating modified nucleoside triphosphates into selections [29]. Our selections with 2′-fluoropyrimidines yielded a relatively small number of nuclease-stable aptamers that appeared to be capable of binding the monomer. The extracellular domain of EGFR contains four subdomains, I, II, III, and IV, with subdomains II and IV being cysteine-rich and therefore also known as CR1 and CR2 [30]. Since binding of the aptamer to both the protein *in vitro* and to cells was insensitive to DTT, we can hypothesize that the epitope bound by the aptamer was distinct from the cysteine-rich subdomains II and IV of the protein, because these contain a number of disulfide bonds. Similarly, the crystal structure and other biochemical results show that domains I and III are involved in EGF binding [31]. Because Aptamer E07 successfully competed with EGF for binding to EGFR, we further hypothesize that the binding site for Aptamer E07 should at least partially overlap the EGF binding site on domains I and III.

We also attempted to determine whether Aptamer E07 could bind to the common EGFR deletion variant, EGFRvIII in which residues 6–273 from domain I (residues 1–165) and II (residues 166–310) are removed. The aptamer showed significant binding relative to no protein controls (**Figure S3 A**). It is therefore possible that Aptamer E07's major binding site on EGFR resides on domain III. Given these promising results, we then assayed whether the aptamer bound to a cell line (U87MG delta vIII) that had been engineered to overexpress the variant EGFRvIII (although this line also still displays low levels of wild-type EGFR [32], [33]). Strong binding and internalization was observed (**Figure S3 B**), indicating that the aptamer can recognize the deletion variant and may promote internalization of this receptor.

Aptamer binding to domains I and / or III is also consistent with the ability of the aptamer to inhibit EGFR function. EGFR is known to be present on the surface of cells in two conformations: an inactive conformation where domain II and IV are tethered, and subdomains I and III are held too far apart for EGF to bind both domains simultaneously, and an active conformation in which domain I becomes available for ligand cobinding with domain III. It has been postulated that about 3–15% of the unstimulated receptor is in the active form at any time, and that EGF binding drives the conformational equilibrium toward the active state [34]. In the active state the dimerization arm of domain II is released from its tether, allowing the protein to homo- or heterodimerize. Subsequent activation of EGFR's intrinsic protein tyrosine kinase activity occurs and leads to autophosphorylation of tyrosine residues in the C-terminal domain. Autophosphorylation in turn triggers a complex intracellular signal transduction pathway involving the Ras-Raf-MAP-kinase cascade, PI3K (phosphatidyl inositol 3-kinase), the downstream protein kinase Akt, and various transcription factors such as STAT (signal transducer and activator of transcription) [35], [36]. These signaling proteins modulate phenotypes such as cell migration, adhesion, invasion, cell proliferation, angiogenesis, and resistance to apoptosis [36]. As a result, EGFR has been shown to be a tumor biomarker [37], and there are a number of already approved anti-EGFR pharmaceuticals with more in clinical trials.

While therapeutic aptamers are virtually unknown at the current time, therapeutic antibodies are widespread [38]. Because of the importance of EGFR in oncogenesis, anti-EGFR antibodies have been developed as therapeutics. A mouse monoclonal anti-EGFR antibody (clone 225) binds to EGFR with affinity similar to EGF (1nM), blocks EGF-induced activation of EGFR tyrosine phosphorylation, and induces internalization of EGFR without stimulating EGFR phosphorylation [39]. To reduce the immunogenicity of mouse antibody C225, a chimera consisting of its murine Fv region and human IgG1 heavy and kappa light chain regions has been developed [23]. The chimeric anti-EGFR antibody, also known as Cetuximab, was approved by the FDA for the treatment of colorectal cancer and head and neck cancer in 2004. Other anti-EGFR antibodies that are in clinical use and that have similar (thought not identical) mechanisms of action include Panitumumab.

Despite the fact that Cetuximab and other antibodies have proven to be clinically useful, they do possess some disadvantages. There are numerous side-effects from treatment with anti-EGFR antibodies, including immunogenic responses such as skin (acneiform rash) and other toxicities that may stem directly from anti-EGFR activity [40], and anaphylactic or allergic reactions [41]. Early trials with head and neck squamous cell carcinomas had to be canceled because of adverse effects, including several deaths. Similar problems were observed during the treatment of non-small-cell lung cancers [42].

In addition, many tumors are inherently resistant to or become resistant to anti-EGFR antibodies [43]. Cetuximab and other many other anti-EGFR antibodies block dimerization [44]. Resistance can arise because of mutations that favor the overexpression of EGFR, the ‘untethered’ conformation, and / or ligand-independent activation [45], [46]. Further, there are reports that there is wide variation in the efficacy of Cetuximab for treating cell lines and cancers that express EGFRvIII [47], [48]. Anti-EGFR aptamers are likely to have lower immunogenicity (and hence potentially lower toxicity) than antibodies, and will interact differently with EGFR than antibodies, potentially increasing efficacy and overcoming resistance to antibody therapeutics.

Our anti-EGFR aptamers may also prove useful as escorts for other therapies. Aptamers have previously been used to escort toxins [49], small organic drugs [50], and even siRNA molecules [8], [11], [51] into cells. Upon binding, both EGF and Cetuximab induce EGFR internalization (although the mechanism of antibody-induced internalization remains unclear) [52]. The internalization of ^125^I-EGF and ^125^I-225 mAb has been compared in A549 lung adenocarcinoma cells [53], and within approximately 15 min, the internal-to-surface ratio was found to plateau at values of 2.5 for ^125^I-EGF and 0.4 for ^125^I-225 mAb. While we are still exploring the mechanism of the anti-EGFR aptamer internalization, we have found there is about 22% of Aptamer E07 internalized into A431 cells within 30 min. This value translates to an internal-to-surface ratio of 0.3, comparable to that found with ^125^I-225 mAb (0.4). We can now explore whether E07 can be further improved by acting as a cytotoxic delivery reagent, including to cells expressing the EGFRvIII deletion variant.

## Supporting Information

Figure S1 **Predicted secondary structure of Aptamer E01 and Aptamer E07**. Secondary structures were predicted using the program MFOLD. The sequence substitutions U40G and C67A were highlighted in red in Aptamer E07.
(TIF)

Figure S2 **Impact of dephosphorylation on aptamer inhibition of EGF-binding.** Alexa 488-labeled EGF (0.1 ug/ml, ca. 1.5 nM) was incubated with A431 cells (green line), and binding assessed by FACS. The interaction could be blocked by 1 uM Aptamer E07 (cyan line) and dephosphorylated Aptamer E07 (dark blue line), but not by a Mutant Aptamer (pink line) or the dephosphorylated Mutant Aptamer (orange line). Counts represent number of cells counted.
(TIF)

Figure S3 **Binding and internalization of anti-EGFR Aptamer E07 in cells expressing the EGFRvIII deletion variant (A) and binding of E07 Aptamer to the EGFRvIII deletion variant protein (B).** (A) Phycoerythrin-labeled Aptamer E07 (100 nM, cyan line) was incubated with U87MG delta vIII cells at 37°C for 30 min. After the binding reaction, cells were exposed to Riboshredder for 10 min at 25°C (pink and orange lines, respectively). Residual fluorescence was analyzed by FACS. Counts represent number of cells counted. (B) Binding was measured were using 0.1 nM aptamer and 50 ug of hEGFR or hEGFRvIII. Binding assays were carried out in triplicate and the average values and standard deviations are shown.
(TIF)

Table S1 Sequences of anti-EGFR aptamers isolated from the N62 pool. Only the random sequence portions of the aptamers are shown. The known Fc-binding motif GGUGCU was highlighted in red. N represents an undetermined nucleotide. The number of times the aptamer was isolated is shown on the right.
(XLS)

Table S2 Sequences of anti-EGFR aptamers isolated from a doped Aptamer E01 pool. Only the random sequence portions of the aptamers are shown. Aptamer E30 and E39 (red) appeared twice. N represents an undetermined nucleotide.
(XLS)
