# Supplementary figures and images for: Inhibition of Cell Proliferation by an Anti-EGFR Aptamer
Source: PLoS One. 2011 Jun 8;6(6):e20299. doi: 10.1371/journal.pone.0020299 (PMC3110755; doi:10.1371/journal.pone.0020299)

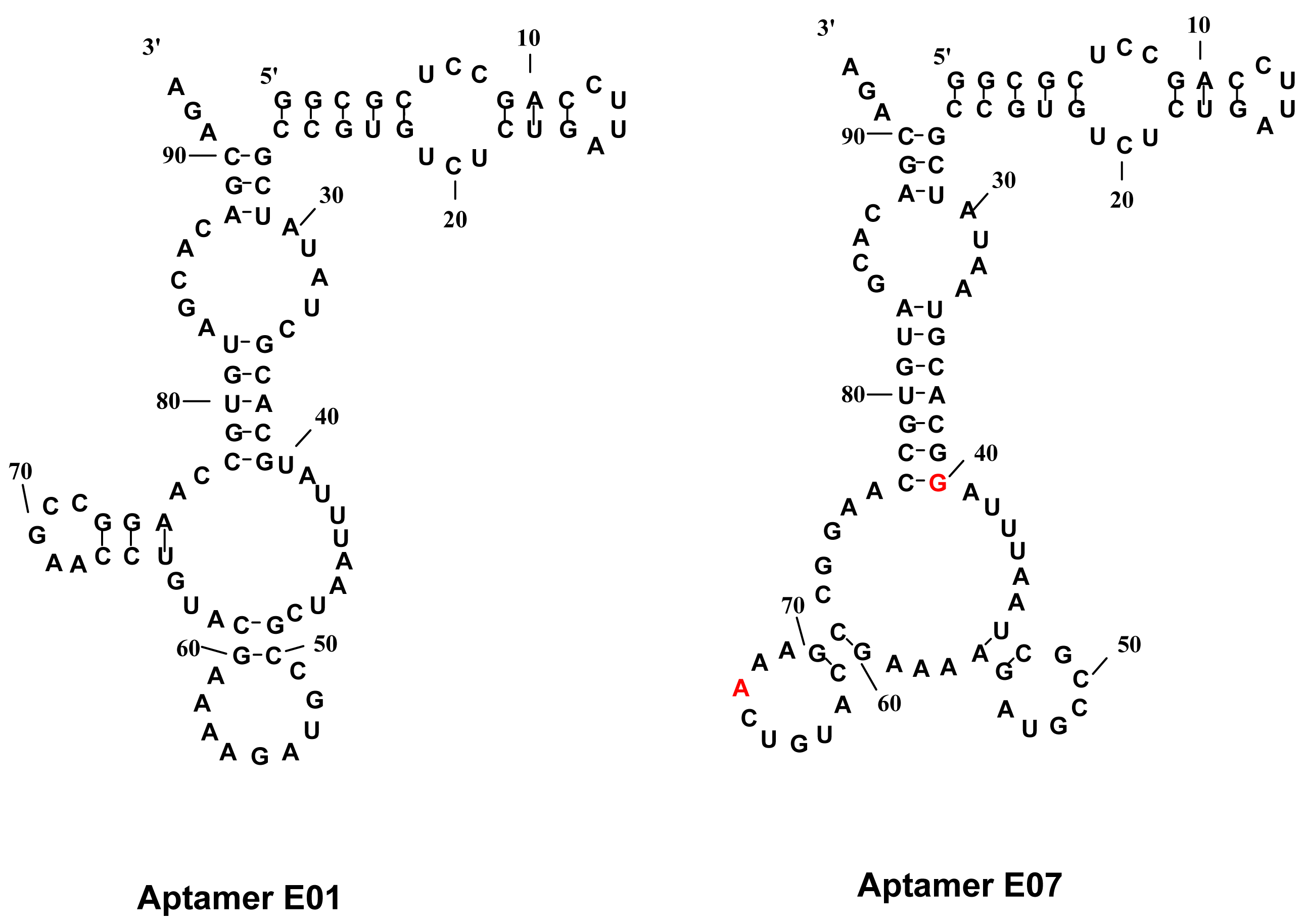

Supplement: Figure S1 — Predicted secondary structure of Aptamer E01 and Aptamer E07. Secondary structures were predicted using the program MFOLD. The sequence substitutions U40G and C67A were highlighted in red in Aptamer E07. (TIF) [file pone.0020299.s001.tif]

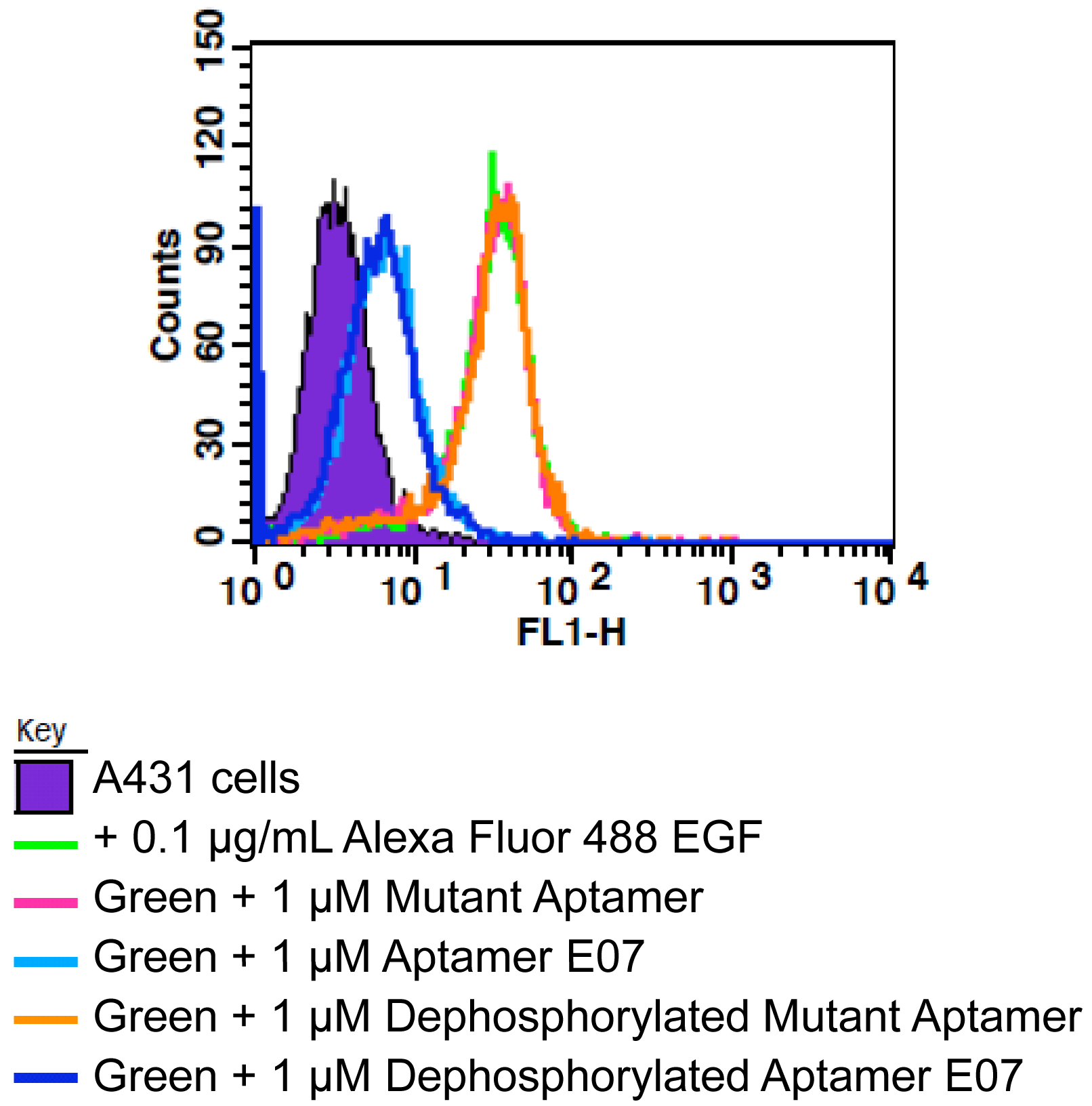

Supplement: Figure S2 — Impact of dephosphorylation on aptamer inhibition of EGF-binding. Alexa 488-labeled EGF (0.1 ug/ml, ca. 1.5 nM) was incubated with A431 cells (green line), and binding assessed by FACS. The interaction could be blocked by 1 uM Aptamer E07 (cyan line) and dephosphorylated Aptamer E07 (dark blue line), but not by a Mutant Aptamer (pink line) or the dephosphorylated Mutant Aptamer (orange line). Counts represent number of cells counted. (TIF) [file pone.0020299.s002.tif]

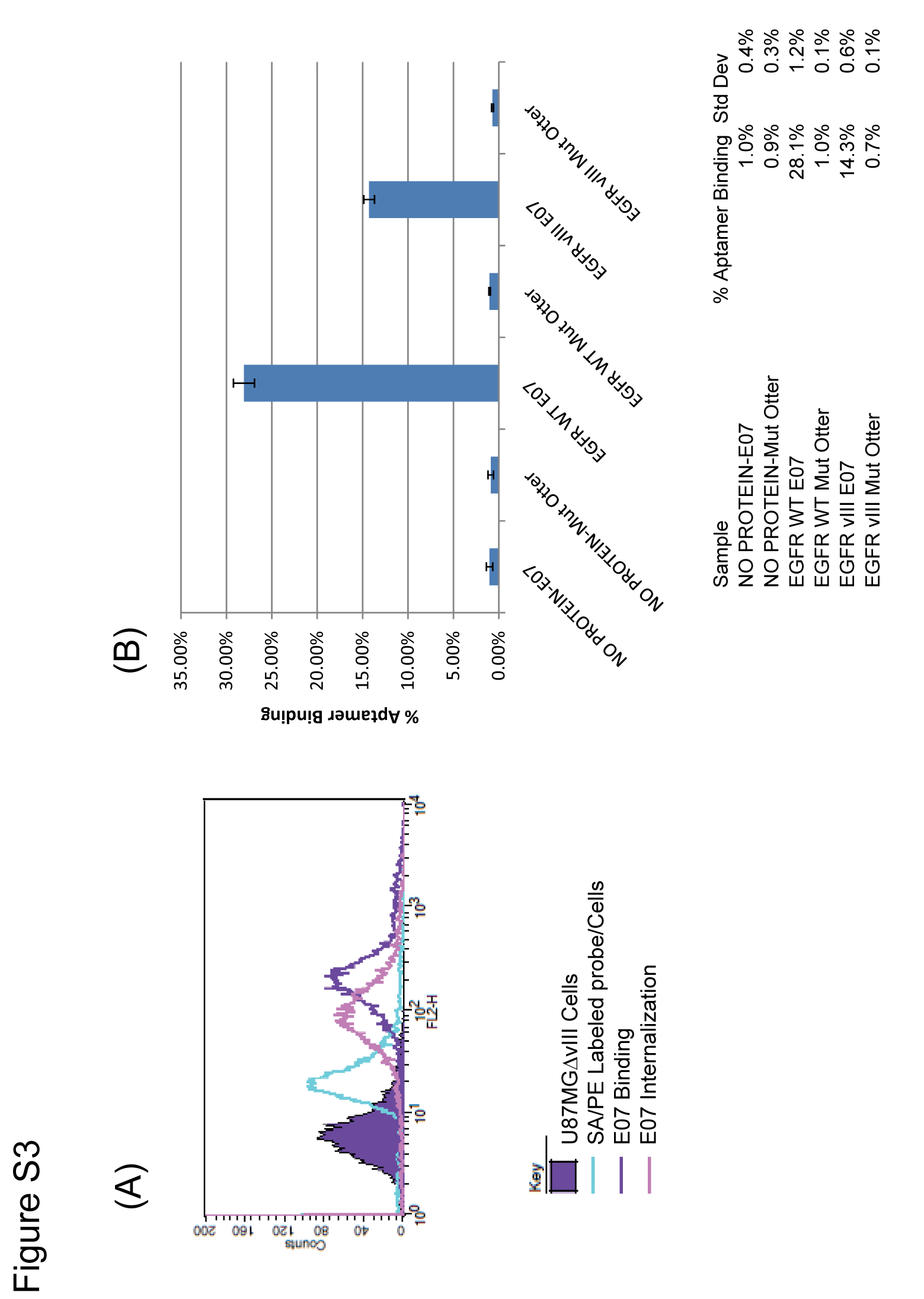

Supplement: Figure S3 — Binding and internalization of anti-EGFR Aptamer E07 in cells expressing the EGFRvIII deletion variant (A) and binding of E07 Aptamer to the EGFRvIII deletion variant protein (B). (A) Phycoerythrin-labeled Aptamer E07 (100 nM, cyan line) was incubated with U87MG delta vIII cells at 37°C for 30 min. After the binding reaction, cells were exposed to Riboshredder for 10 min at 25°C (pink and orange lines, respectively). Residual fluorescence was analyzed by FACS. Counts represent number of cells counted. (B) Binding was measured were using 0.1 nM aptamer and 50 ug of hEGFR or hEGFRvIII. Binding assays were carried out in triplicate and the average values and standard deviations are shown. (TIF) [file pone.0020299.s003.tif]
